# Slow time perception can be learned

**DOI:** 10.3389/fpsyg.2014.00209

**Published:** 2014-03-12

**Authors:** Ralf Buckley

**Affiliations:** International Chair in Ecotourism Research, School of Environment, Griffith UniversityGold Coast, QLD, Australia

**Keywords:** risk, recreation, cognition, fear, dilation, accidents

Arstila (2012) reviewed competing hypotheses for the perception of slow time. Perception of time is fundamental to human understanding, and the neurophysiological mechanisms involved are heavily studied (Eagleman, 2008; Wittmann, 2011, 2013; Phillips, 2013). Abnormal perceptions such as slow time provide an analytical tool. Here I put forward additional evidence from high-risk, high-skill outdoor recreation.

Reported experiences of slow time are commonly associated with accidents which are sudden, unexpected, and immediately life-threatening; and they also involve a perception of greatly increased speed and clarity of mental processes (Arstila, 2012). Current hypotheses (Stetson et al., 2007; Eagleman, 2008; Arstila, 2012; Phillips, 2013) are that under extreme stress, either (1) our senses record data at higher density; (2) our brains sample more of these data; (3) our brains process these sampled data faster; or (4) our memories store data at higher density. Arstila (2012) and Phillips (2013) dismiss (1) and (4) using various lines of evidence. They argue for (3) and possibly also (2). Arstila (2012) hypothesizes a physiological mechanism, very rapid activation of the locus coeruleus-norepinephrine, LC-NE (Aston-Jones and Cohen, 2005). Phillips (2013) suggests that our brains use the rate of subconscious (“non-perceptual”) mental processes as a clock. If this processing accelerates, our time perception changes.

Previous analyses rely ultimately on two types of primary data, both subject to shortcomings (Arstila, 2012; Phillips, 2013). The first, compiled by Noyes and Kletti (1972) are second-hand reports from people involved in accidents. The second, exemplified by Stetson et al. (2007), use experimental approaches such as free-fall into a net. These may be frightening, but are neither surprising nor life threatening. Many high-skill outdoor recreation activities, in contrast, do involve immediate threat of death. Examples include: cliff take-offs in hang-gliders, kayaking waterfalls or Class V rapids, and surfing big hollow waves breaking on shallow reefs. Here I draw on four decades' ethnographic experience in these activities, including >30000 participant-hours direct observation of practitioner behavior and emotions, multiple face-to-face and electronic communications with 20 highly skilled exponents, and 60 autoethnographic analyses of narrow-escape incidents. These data were compiled during research on fear, thrill, and rush in risk recreation (Buckley, 2012). Autoethnographic data are derived from direct observation, and can be examined in greater depth than alloethnographic reports (Anderson, 2006). Triangulation to accident literature is provided by autoethnographic data from 3 car crashes, including injury and momentary belief of death.

These data yield three findings. The first is that individuals are capable of accelerated physical action as well as accelerated mental processing. This includes extremely fine and fast physical adjustments to control body and equipment very powerfully and precisely, in response to equally fast and detailed perceptions of one's own body and the surrounding environment (Buckley, 2012). This matches reports from climbers in accidental falls (Noyes and Kletti, 1972; Arstila, 2012), that individuals “acted with lightning-quickness in accord with accurate judgment.”

The second finding is that slow-time perception and action can be learned unconsciously, through experience and training. Skilled surfers can adjust their boards and bodies in freefall takeoff on large steep hollow waves at exactly the angle to ride the wave as the lip barrels over their head. Skilled kayakers can adjust boat, body, and paddle so as to take exactly the one survivable line through rapids and over waterfalls. Less skilled participants perceive only confusion, and are likely to freeze, panic, or act in ways which increase danger (Buckley, 2012). It is also experience and training which allow skilled exponents of many physical arts to achieve feats which appear impossibly fast and precise. There are many examples in ball and boardsports, gymnastics, acrobatics, dance, martial arts, archery, shooting, swordsmanship, and in aircraft, car and motorbike racing and stunt driving. The third finding is that at least to some degree, some trained individuals can actively turn on slow-time perception and processing. This is part of the process of “pumping up” used by skilled athletes preparing for a difficult performance.

This evidence supports the suggestion by Arstila (2012) that humans possess a specialized hormonal or neurophysiological mechanism for high-speed cognition; and that this is activated inadvertently by real fear of imminent violent death in accidents, emergencies, and certain extreme sports; and may on some occasions be activated intentionally by individuals who have trained themselves to do so. This raises three questions. Firstly, why is this mechanism only turned on occasionally? Presumably, it has high metabolic costs or side effects which reduce biological fitness if it is activated continually. Secondly, if some individuals have learned to activate slow-time perception, processing and action, what is the mechanism? Thirdly, some extreme-sports practitioners report moments not of high-speed mental processing, but of complete calm and mental clarity at instants of highest danger. Such moments, once experienced, are effectively equivalent to self-perceived religious experiences, and highly sought after. How are these two diametrically opposed perceptions connected?
